# Additively Manufactured 3D Micro-bioelectrodes for Enhanced Bioelectrocatalytic Operation

**DOI:** 10.1021/acsami.2c20262

**Published:** 2023-03-10

**Authors:** Keyvan Jodeiri, Aleksandra Foerster, Gustavo F. Trindade, Jisun Im, Diego Carballares, Roberto Fernández-Lafuente, Marcos Pita, Antonio L. De Lacey, Christopher D Parmenter, Christopher Tuck

**Affiliations:** † Centre for Additive Manufacturing, Faculty of Engineering, University of Nottingham, University Park, Nottingham NG7 2RD, United Kingdom; ‡ National Physical Laboratory, Hampton Road, Teddington TW11 0LW, United Kingdom; § Instituto de Catálisis y Petroleoquímica, CSIC, C/Marie Curie 2, 28049 Cantoblanco, Madrid, Spain; ∥ Center of Excellence in Bionanoscience Research, Member of the External Scientific Advisory Board, King Abdulaziz University, 21589 Jeddah, Saudi Arabia; ⊥ Nanoscale and Microscale Research Centre, University of Nottingham, University Park, Nottingham NG7 2RD, United Kingdom

**Keywords:** additive manufacturing, microelectrodes, surface functionalization, electroless metal plating, enzymatic fuel cells

## Abstract

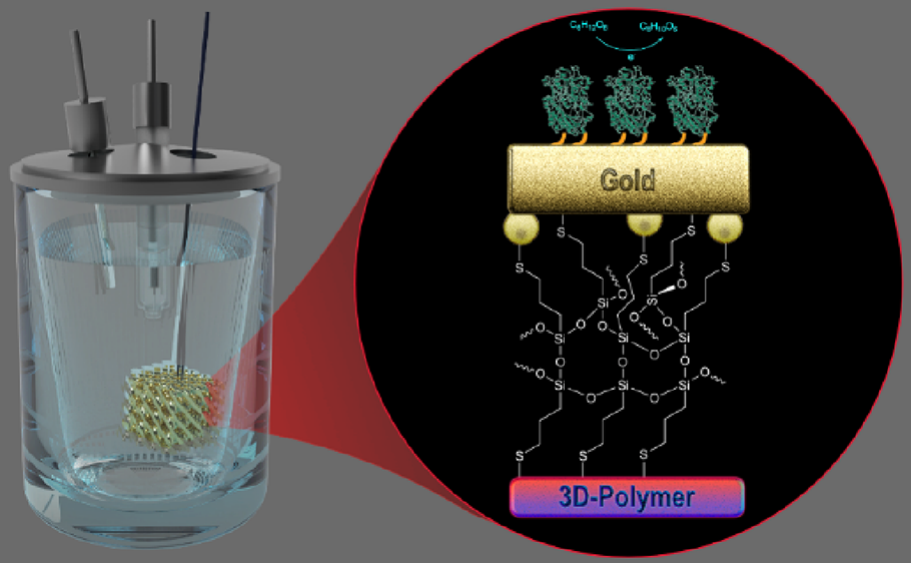

The drive toward miniaturization of enzyme-based bioelectronics established a need for three-dimensional (3D) microstructured electrodes, which are difficult to implement using conventional manufacturing processes. Additive manufacturing coupled with electroless metal plating enables the production of 3D conductive microarchitectures with high surface area for potential applications in such devices. However, interfacial delamination between the metal layer and the polymer structure is a major reliability concern, which leads to device performance degradation and eventually device failure. This work demonstrates a method to produce a highly conductive and robust metal layer on a 3D printed polymer microstructure with strong adhesion by introducing an interfacial adhesion layer. Prior to 3D printing, multifunctional acrylate monomers with alkoxysilane (−Si–(OCH_3_)_3_) were synthesized via the thiol–Michael addition reaction between pentaerythritol tetraacrylate (PETA) and 3-mercaptopropyltrimethoxysilane (MPTMS) with a 1:1 stoichiometric ratio. Alkoxysilane functionality remains intact during photopolymerization in a projection micro-stereolithography (PμSLA) system and is utilized for the sol–gel reaction with MPTMS during postfunctionalization of the 3D printed microstructure to build an interfacial adhesion layer. This leads to the implementation of abundant thiol functional groups on the surface of the 3D printed microstructure, which can act as a strong binding site for gold during electroless plating to improve interfacial adhesion. The 3D conductive microelectrode prepared by this technique exhibited excellent conductivity of 2.2 × 10^7^ S/m (53% of bulk gold) with strong adhesion between a gold layer and a polymer structure even after harsh sonication and an adhesion tape test. As a proof-of-concept, we examined the 3D gold diamond lattice microelectrode modified with glucose oxidase as a bioanode for a single enzymatic biofuel cell. The lattice-structured enzymatic electrode with high catalytic surface area was able to generate a current density of 2.5 μA/cm^2^ at 0.35 V, which is an about 10 times increase in current output compared to a cube-shaped microelectrode.

## 1 Introduction

The drive toward the development of enzyme-based bioelectronics, such as biofuel cells and biosensors, raises a corresponding need for highly conductive, chemically stable, and easily functionalizable electrodes. For instance, it has been shown that a 3D microstructure with high surface area provides more catalytically active surface sites and increases the efficiency of electrocatalytic reactions and current output in enzymatic biofuel cells (EFCs)^1,2^ and the sensitivity of electrochemical sensing.^3−5^ However, the fabrication of conductive electrodes with high surface area-to-volume ratio and highly complex structures is challenging with conventional manufacturing processes.

The advancement in additive manufacturing (AM), also known as 3D printing, enables the fabrication of 3D customized microstructures with almost any arbitrary design. Various AM methods such as extrusion,^6−8^ powder bed fusion,^9^ vat photopolymerization,^10^ and inkjet printing^11^ were used to fabricate 3D microelectrodes. However, these methods utilized materials containing conductive constituents such as metal nanoparticles to fabricate 3D microelectrodes, which require a high-temperature sintering process. The conductivity achieved by those methods was a few orders of magnitude lower than that of the counterpart bulk metal.^12−14^

Electroless plating (EP) on additively manufactured 3D structures offers the potential to deposit a broad range of metals on 3D polymeric templates^12,13,15,16^ and produce a continuous and uniform metal layer with high electrical conductivity.^17^ However, a significant challenge of this method is interfacial delamination between the deposited metal layer and polymer scaffold due to poor adhesion, which impacts on device performance and reliability. Different techniques have been developed to ensure good adhesion of the metal layer to the polymer surface. These include physical and chemical procedures such as chemical etching,^16,18^ plasma treatment,^19^ and UV treatment^20^ before the electroless plating process. However, the challenges still remain: (i) if the adhesion of the adsorbed metallic coating is not strong enough, it will flake off once the plating procedure is complete,^16,21^ and (ii) the interior surfaces of 3D objects with complicated geometries remain untreated using UV and plasma due to line-of-sight restrictions.^22,23^

**Figure 1:**
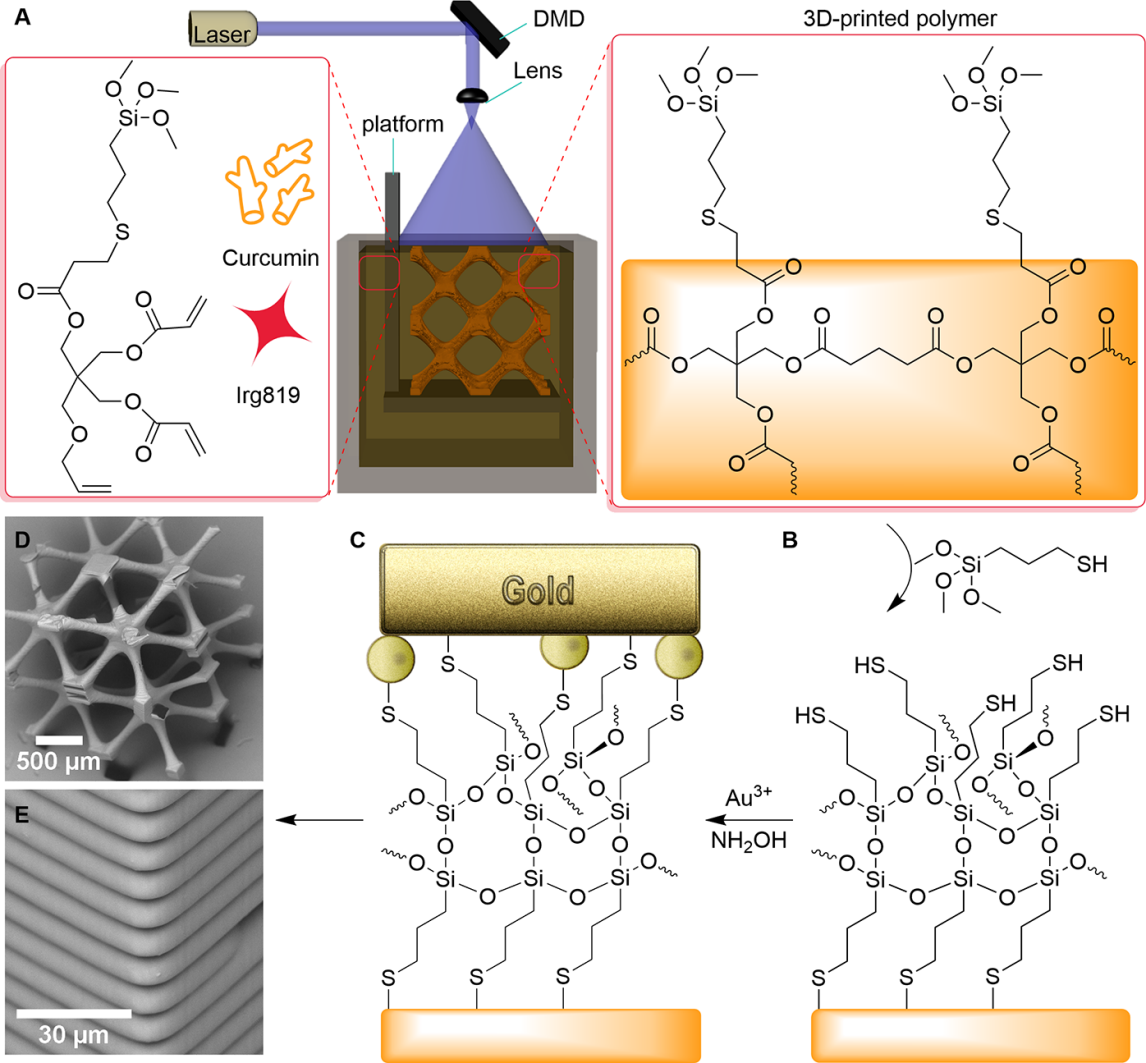
Fabrication of robust 3D conductive microstructures: (A) 3D printing of a functional photocurable resin using PμSLA, (B) introduction of an interfacial adhesion layer by surface functionalization with MPTMS, and (C) electroless gold plating of a 3D printed polymer microstructure. (D) SEM image of the 3D printed BCC lattice structure. (E) Magnified SEM image of 3D printed BCC showing the thickness of a single printed layer.

In this paper, we present a method to produce highly conductive and robust 3D gold microelectrodes by introducing an interfacial adhesion layer between the metal coating and the 3D printed polymer scaffold. Our method of manufacturing 3D printed conductive microstructures consists of three simple steps: (i) 3D printing of a polymer scaffold using a functional photocurable resin, (ii) surface functionalization to build an interfacial adhesion layer, and (iii) electroless gold plating. Various 3D model microstructures with varying surface area are fabricated using projection micro-stereolithography (PμSLA) capable of printing objects down to a 2 μm resolution. The interfacial adhesion layer is introduced by the design of a photocurable resin for a 3D polymer scaffold and consecutive surface functionalization with 3-mercaptopropyltrimethoxysilane (MPTMS) (Figure 1A). MPTMS is selected for the interfacial adhesion layer because it can be covalently bonded to the surface of the 3D printed polymer via hydrolysis and condensation of silanol. The thiol group on the other end of MPTMS can form a covalent bonding with gold atoms via gold-thiolate during electroless gold plating in the third step. This interface engineering allows the uniform gold deposition due to the gold–thiol interaction at the interface, which results in a high conductivity of 2.2 × 10^7^ S/m (53% of bulk gold conductivity) and strong interfacial adhesion.

As a proof-of-concept, we examine the use of a 3D gold microelectrode as an enzymatic anode for a single enzymatic biofuel cell (EFC). Glucose oxidase (GOx) is utilized as a model enzyme because it is often used in the production of EFCs.^24,25^ In the preliminary test, a diamond lattice gold microelectrode with high catalytic surface area showed a significant increase in the current output compared to the one in the simple cube form, which shows the great potential of 3D conductive microarchitectures for applications such as biofuel cells and biosensors.

## 2 Results and Discussion

### 2.1 Preparation of the Functional Photocurable Resin

Our strategy to improve the interfacial adhesion between a polymer scaffold and the deposited metal is to functionalize the surface of a 3D printed microstructure with thiol functionality using MPTMS that can be chemically bonded to gold during electroless plating (Figure 1A). For that, the photocurable resin was designed to provide alkoxysilane functionality on the printed surface that can be utilized for the surface functionalization reaction with MPTMS. First, the multifunctional acrylate monomer with alkoxysilane (MP monomer) was synthesized using the thiol–Michael addition reaction between pentaerythritol tetraacrylate (PETA) and MPTMS with a 1:1 stoichiometric ratio (Figure S1).^26^ Three acrylates and one alkoxysilane functionalities of the MP monomer were verified by ^1^H and ^13^C nuclear magnetic resonance (NMR) (Experimental Section). The photocurable resin for PμSLA was prepared by mixing MP monomers with the photoinitiator (Irgacure 819) and photoabsorber (curcumin). All resin components including Irgacure 819,^27^ curcumin,^28^ PETA,^29^ and MPTMS^30^ are proven to be biocompatible, which offer the potential use of 3D printed microstructures for bioelectronic devices such as implantable biofuel cells.^27,31,32^ The optimal concentration of Irgacure 819 to achieve the highest degree of polymerization (DP) was determined to be 1 wt % using Fourier transform infrared (FTIR) spectroscopy at different concentrations ranging from 0.5 to 1.75 wt %. The DP, calculated from the peak areas of the unsaturated C=C bond at 1650 cm^–1^ and C=O bond at 1730 cm^–1^ of uncured resin and cured polymers (MP polymer), increased with the concentration of Irgacure 819 up to 1 wt % and then decreased at higher concentrations (Figure S1B). This might be due to the rapid and high production of free radicals and the rapid reaction with monomers that competes with polymerization.^33^ The photocurable functional monomer was then mixed with a photoabsorber to prevent overpolymerization (OP) and increase lateral and vertical printing resolution by controlling light scattering and penetration. The optimal concentration of curcumin was determined by calculating the OP of the printed bridge-shaped model structure (Figure S2).^34^ The OP of the printed bridge made with a resin containing 0.5 wt % curcumin was calculated to be as low as 7.60%, showing high dimensional printing accuracy.

### 2.2 3D Printing of Polymer Microstructures

The optimized photocurable resin, including 1 wt % of Irgacure 819 and 0.5 wt % of curcumin, was used to print a 3D polymer microstructure using PμSLA as a platform for electroless gold plating. The PμSLA allows for high-quality printing with a nominal resolution of 2 μm in the *XY* direction and 5 μm in the *Z* direction. The angular precision of the printed sample was investigated using a body-centered cubic (BCC) lattice structure by comparing the interaxial angles of the designed and printed lattice structures (Figure S3C,D). The BCC lattice was chosen as a model structure to check the printing quality of the functional ink formulation and printing parameters for PμSLA because it consists of all the necessary geometries such as pores, small struts, and overhangs that are parts to create complex structures with a large surface area. Figure 1D depicts a printed lattice structure with a layer thickness of 10 μm (Figure 1E). The lateral printing resolution and angular precision in all directions were calculated to be 88 and 82.5%, respectively, indicating high spatial resolution (Figure 1D and Figure S2). X-ray photoelectron spectroscopy (XPS) analysis on the 3D printed cube (Figure S3A) polymer (MP polymer) revealed the characteristic Si2p peak at 102 eV and O1s peak at 532 eV for alkoxysilane (−Si–(OCH_3_)_3_) groups and S2p peak at 163.8 eV for the thioether (C–S) bond, confirming the successful implementation of alkoxysilane groups on the polymer surface (Figure S4). These alkoxysilane groups take part in the sol–gel reaction with MPTMS in the next step to build the interfacial adhesion layer for gold deposition.

### 2.3 Thiol Functionalization of the 3D Printed Microstructure

**Figure 2:**
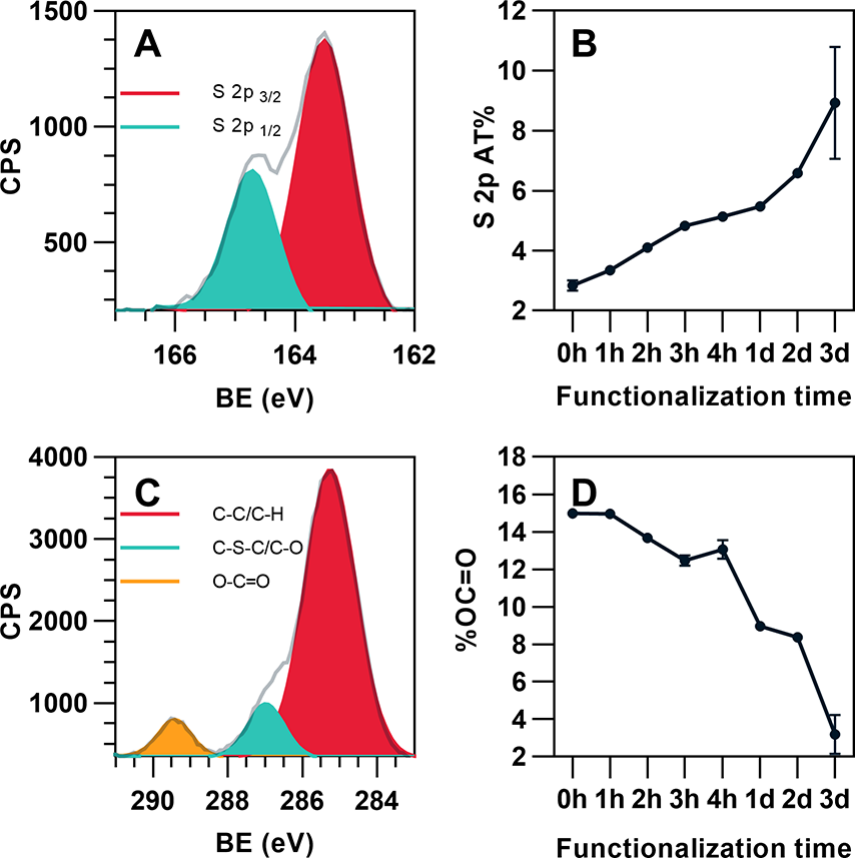
XPS analysis of the 3D printed polymer with thiol functionalization (SG-MP polymer). (A) High-resolution S2p core-level spectrum of the SG-MP polymer prepared at *t*_func_ = 3 h. (B) Atomic percentage of S2p of the SG-MP polymer after thiol functionalization at different *t*_func_. (C) High-resolution C1s core-level spectrum of the SG-MP polymer prepared at *t*_func_ = 3 h. (D) The proportion of teh O–C=O moiety to other C1s chemical states of the SG-MP polymer at different *t*_func_. The error bars represent the standard deviation of at least three separate tests.

An interfacial adhesion layer with thiol functionality was introduced by the sol–gel reaction of MPTMS with alkoxysilane groups on the surface of the 3D printed microstructure. The sol–gel reaction was carried out for different reaction times (*t*_func_) ranging from 1 h to 3 days to find the optimal functionalization reaction time, and the amount of thiol groups was estimated using XPS analysis on the samples before and after the sol–gel reaction. The atomic percentages of S and Si relative to C of the surface functionalized polymer (SG-MP polymer) after the sol–gel reaction at *t*_func_ = 3 days were increased by three times compared to those for the MP polymer (*t*_func_ = 0 h), confirming the successful formation of the thiol adhesion layer (Table S1). The high-resolution S2p core-level spectrum for the SG-MP polymer shows the peak at 163.8 eV assigned for free thiols, and no oxidized sulfur was found (Figure 2A). The atomic percentage of S2p for free thiols increases with *t*_func_, confirming the formation of a thicker interfacial adhesion layer at longer *t*_func_ (Figure 2B). The high-resolution C1s spectrum of the SG-MP polymer also supports the formation of the thiol adhesion layer. The C1s peak in Figure 2C was resolved by peak fitting into three chemical states: C–C at 285.0 eV, C–S/C–O at 287.0 eV, and O–C=O at 289.0 eV. The peak at 289.0 eV is assigned to be O–C=O moieties of PETA from the MP polymer,^35^ and reduction of this peak after sol–gel reaction indicates the introduction of a thiol adhesion layer on the surface of the MP polymer (Figure 2D).

The successful introduction of the thiol adhesion layer was also supported by time-of-flight secondary ion mass spectrometry (ToF-SIMS) analysis. The SH^–^ signal (at 32.98 u) for the SG-MP polymer was enhanced compared to the MP polymer shown in ToF-SIMS spectra (Figure S5A). The C_3_H_3_O^–^ peak (at 55.02 u) corresponding to the methoxy group was significantly decreased after the sol–gel reaction, which might be attributed to the hydrolysis of the methoxy group during the reaction (Figure S5A). Furthermore, the intensity of the SH^–^ signal shown in ToF-SIMS mapping images confirms that free thiols are distributed evenly throughout the surface (Figure S5BC).

### 2.4 Electroless Gold Plating

The continuous gold layer was deposited on the 3D polymer microstructure (SG-MP polymer) by the following steps: (i) seeding with gold nanoparticles (AuNPs) and (ii) electroless gold plating by reduction of the gold precursor to Au(0). AuNPs can be attached to the thiol-functionalized adhesion layer during the seeding step. The AuNP-seeded polymer was then soaked in an electroless plating solution containing a gold precursor (HAuCl_4_) and a reducing agent (hydroxylamine) and sonicated for 7 min for continuous gold growth on the surface. These AuNPs on the surface act as a catalyst in the electroless plating reaction and help to reduce Au^3+^ to Au(0) by hydroxylamine preferentially on the AuNP-seeded sites. In addition, during the plating procedure, the thiol groups on the surface of the SG-MP polymer are deprotonated in the plating solution and interact strongly with Au^3+^, resulting in a high concentration of Au^3+^ on the polymer surface for continuous gold growth and thus the formation of a uniform gold layer on the surface.^36−39^

**Figure 3:**
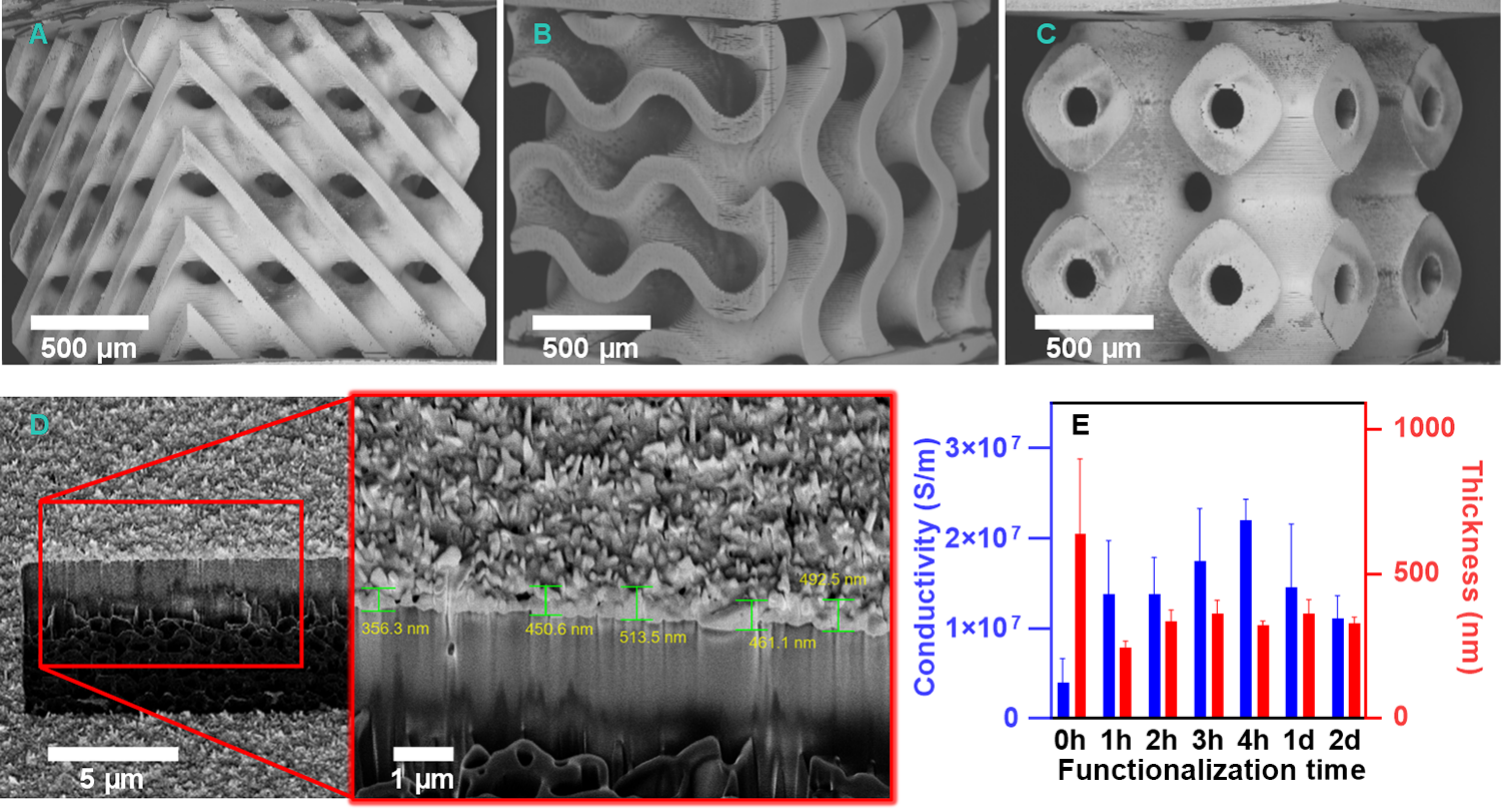
SEM images of electroless gold-plated 3D microstructures: (A) diamond, (B) gyroid, and (C) primitive. (D) FIB-SEM analysis on the cross section of the electroless plated sample prepared from the SG-MP polymer at *t*_func_ = 3 h. Inset shows the magnified image showing the average gold thickness of 456 ± 49 nm. (E) Effect of thiol functionalization time on the electrical conductivity of gold deposited samples (cube structure with dimension of 2 × 2 × 0.5 mm). The reference sample (0 h) for conductivity measurement was electroless plated using a gentler mixing method without sonication because the gold did not adhere to the surface during sonication. The error bars show the standard deviation of at least three independent experiments.

Sonication during electroless plating was carried out, unlike conventional electroless plating,^40,41^ because it helps create a more uniform gold coating by preventing the formation of large gold agglomeration weakly bound on the surface. It also helps to remove Cl_2_ bubbles generated during the reaction that prevents the electroless plating solution from penetrating inside the lattice structure. We note that this method only works well with the SG-MP polymer with a thiol interfacial adhesion layer. The MP polymer without thiol groups does not provide strong adhesion between the created gold layer and the polymer surface. Hence, the weakly bound gold on the MP polymer was detached from the surface during sonication, leading to reduced gold coverage on the surface (Figure S6). On the other hand, the thiol interfacial adhesion layer on the SG-MP polymer provides strong binding sites for gold such that sonication only disturbs weakly bound gold and does not affect the deposited gold with strong adhesion. We successfully fabricated 3D gold electrodes of three triply periodic minimal surface (TPMS) lattice structures: diamond, gyroid, and primitive (Figure 3A–C). The electrodes with these lattice structures are particularly interesting for applications requiring large surface areas with ideal stiffness, such as biosensors and compact, lightweight fuel cells with high energy density.^42^ Our methodology ensures the uniform deposition of gold on both the lattice top surface and the inner surface of pores within the lattice proved by SEM and energy-dispersive X-ray analysis (EDX) mapping data (Figure 3A–C and Figures S7 and S8). Minor cracks at the edges of the 3D printed lattice structures are found in Figure 3A–C presumably due to the sonication-induced polymer scaffold damage during electroless gold plating. However, the deposited gold coating layer remained intact because of the strong adhesion between gold and polymer scaffold, and it did not affect the electrical properties of the 3D gold electrodes (Figure S9). Each set of microelectrodes was produced and characterized three times to ensure reproducibility of our methodology.

### 2.5 Electrical Conductivity

The sheet resistance (*R*_s_) and electrical conductivity (σ) of the deposited gold on the 3D printed polymer structure were measured using the Van der Pauw method and calculated from eqs 5 and 6, respectively (Experimental Section). The 3D cube-shaped electrodes with dimension of 2 × 2 × 0.5 mm were fabricated for electrical conductivity and gold layer thickness measurement (Figure 3). The average thickness of the deposited gold layer was evaluated to be 330 ± 60 nm from the cross-sectioned sample using focused ion beam scanning electron microscopy (FIB-SEM) (Figure 3D). We note that the conductivity of the electroless gold plated sample was improved by the introduction of the thiol interfacial adhesion layer and increasing the *t*_func_ (Figure 3E). Because electroless plating on the MP polymer in a sonication mode does not create a gold layer on the surface, we used the sample (*t*_func_ = 0 h) electroless plated in a soft agitation mode for the conductivity measurement. The conductivity (σ = 1.4 × 10^7^ S/m) of the sample with a thiol interfacial adhesion layer (*t*_func_ = 1 h) was 1 order magnitude higher than that (σ = 4 × 10^6^ S/m) of the sample without an adhesion layer (*t*_func_ = 0 h) (Figure 3E). The highest conductivity (σ = 2.2 × 10^7^ S/m, 53% of bulk gold conductivity) of the deposited gold layer was achieved from the sample prepared at *t*_func_ = 4 h. The conductivities of the samples prepared at *t*_func_ = 1 day and *t*_func_ = 2 days were slightly lower compared to that of the sample at *t*_func_ = 4 h. This might be due to the nonuniform, thick layer of MPTMS polymer formed at longer *t*_func_ during surface functionalization,^43^ leading to the formation of a rough gold layer with uneven thickness (Figure S10). The sheet resistance of 3D gold microelectrodes with three TPMS lattice structures (diamond, gyroid, and primitive) was also measured in four-point geometry and calculated using eq 5 (Experimental Section). The slightly higher *R*_s_ from 0.3 to 0.6 Ω/sq for 3D gold diamond, gyroid, and primitive microelectrodes compared to the cube structure (*R_s_* ∼ 0.1 Ω/sq) was obtained presumably because of the 3D curved and complex gold surface structures between four probes (Figure S9).

### 2.6 Interfacial Adhesion

**Figure 4:**
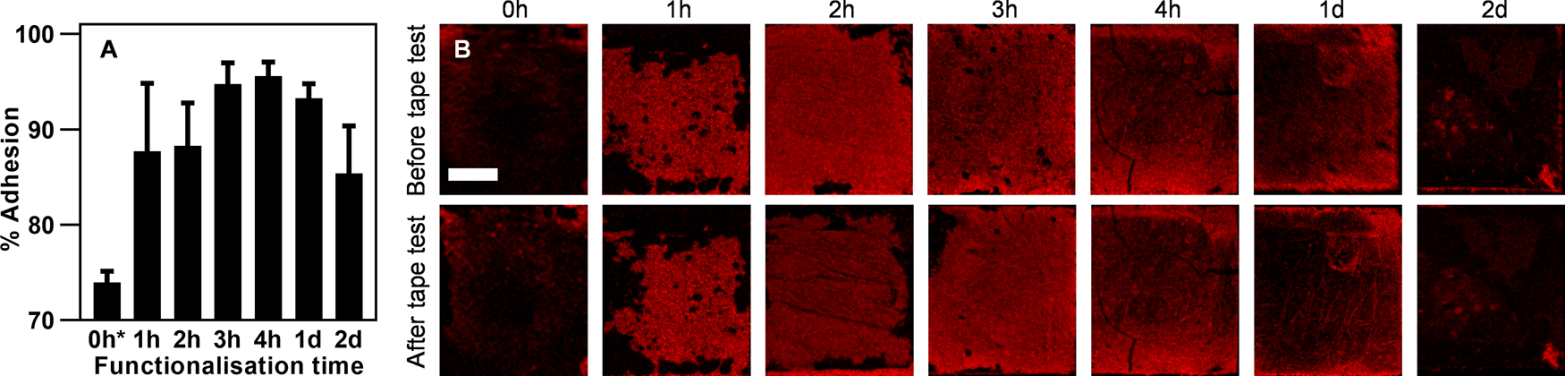
Effect of thiol functionalization on interfacial adhesion between the deposited gold and the polymer surface: (A) the percentage of gold that remained on the polymer surface after adhesion tape test. (B) EDX Au mapping data before and after adhesion tape test. Scale bar represents 500 μm. The error bars show the standard deviation of at least three separate analyses.

The mechanical adhesion between the deposited gold and the polymer scaffold was measured by applying a high-performance clear 3M scotch tape on the electroless gold-plated cube-shaped samples and then peeling it off from the sample at an angle of 90° (estimated peel force between 50 and 250 N/m).^44^ The sample before and after adhesion test was analyzed using SEM coupled with EDX. The adhesion of the deposited gold was quantified by estimating the area of gold from EDX mapping images before and after the adhesion test (eq 4). Without the introduction of the interfacial thiol adhesion layer, only 15 ± 2% of the deposited gold remained on the polymer surface after the tape test, indicating poor adhesion (Figure 4A). However, interfacial adhesion of the deposited gold layer was significantly increased by introducing the thiol adhesion layer shown in Figure 4B.

The maximum interfacial adhesion (97% of gold survived from the adhesion test) was achieved from electroless gold plating of the SG-MP polymer at *t*_func_ = 4 h. We attribute the enhanced adhesion to the strong bonding between thiols and gold at the interface between the deposited gold and the polymer surface. A decrease in the amount of deposited gold and adhesion for the sample at *t*_func_ = 1 and 2 days was observed. This might be due to the delamination of physically adsorbed MPTMS from the surface of the SG-MP polymer during electroless plating in a sonication mode and adhesion test. A similar phenomenon was observed from the adhesion failure of electroless nickel-phosphorous film on the surface of silicon wafer where weakly bound multilayer grafting of alkoxysilane was formed on the silicon wafer.^43^

### 2.7 Interfacial Analysis

We investigated the surface and interfacial chemical composition of the gold-coated 3D microstructure to explore the interfacial bonding between the gold and the printed polymer surface. XPS and EDX analyses were performed on the SG-MP polymers (*t*_func_ = 4 h) before and after electroless gold plating. For the SG-MP polymer without gold coating, the S2p_3/2_ peak appears at 163.8 eV, indicating the existence of the free thiols on the polymer surface (Figure S11A). To confirm the gold-thiolate bonding using XPS, we prepared the sample with a thin gold layer by reducing the electroless plating time from 7 min for thick gold coating (330 ± 60 nm) to 3 min. The S2p core-level spectrum of the SG-MP polymer coated with a thin gold layer exhibits the shift of the S2p_3/2_ peak to 163.2 eV, indicating the gold-thiolate bonding (Figure S11B).^45^ The Au4f_7/2_ peak of the thin gold-coated sample appears at 83.6 eV for Au(0), with a shoulder at 84.2 eV. The shoulder peak at 84.2 eV might be attributed to the discontinuous gold island formation on the polymer surface and charged when the photo-hole is not promptly neutralized (Figure S11C).^46^ This peak disappears for the thick gold-coated sample shown in Figure S11D, indicating the successful formation of the dense and continuous gold film.

**Figure 5:**
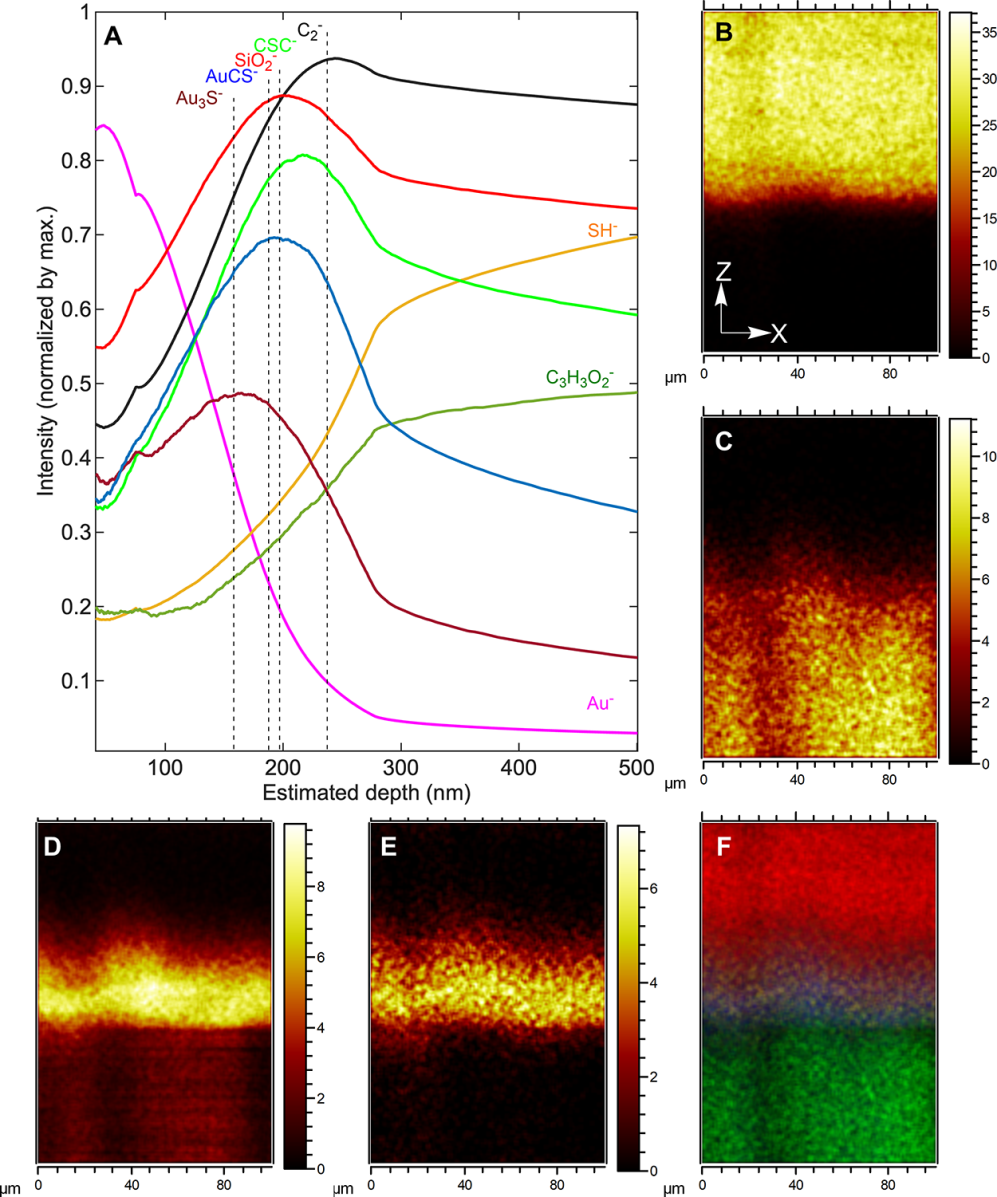
Interfacial chemical composition analysis on gold deposited sample (*t*_func_ = 4 h) using ToF-SIMS analysis. (A) ToF-SIMS depth profiles of the sample. Cross-section maps along the *XZ* direction of (B) Au^3–^, (C) CHO, (D) SiO_2_, and (E) AuS and (F) overlay of the signals for panels B–E (red: Au^3–^, yellow: AuS, blue: SiO_2_, and green: CHO).

ToF-SIMS depth profiling in combination with FIB-SEM was used to investigate the interfacial chemical composition and estimate the thickness and homogeneity of the interfacial adhesion layer of the gold-coated sample. The normalized ToF-SIMS depth profiles of the gold-coated sample (*t*_func_ = 4 h) in Figure 5 show the interfacial adhesion layer consisting of gold-thiolate bonding characterized by Au_3_S^–^ and AuCS^–^ and MPTMS evidenced by the signals of SiO^2–^ and CSC^–^ with an estimated thickness of 50 nm between the deposited gold layer (Au^–^-rich area) and the polymer surface (C_3_H_3_O_2_^–^-abundant region). *XZ* cross-section mapping images reconstructed from the depth profile data in Figure 5B–F reveal the formation of a homogeneous interfacial adhesion layer across the sample.

The successful introduction of the interfacial adhesion layer and strong gold-thiolate interfacial bonding formation during electroless plating ensure the uniform and compact deposition of the gold layer on the polymer surface, leading to high electrical conductivity and strong adhesion. This methodology will allow the simple fabrication of 3D conductive microarchitectures with various design form factors and surface areas and provide the device reliability and performance because of strong adhesion, offering a great potential for novel electronics, including sensors and energy devices.

### 2.8 3D Microelectrode as an Enzymatic Anode

**Figure 6:**
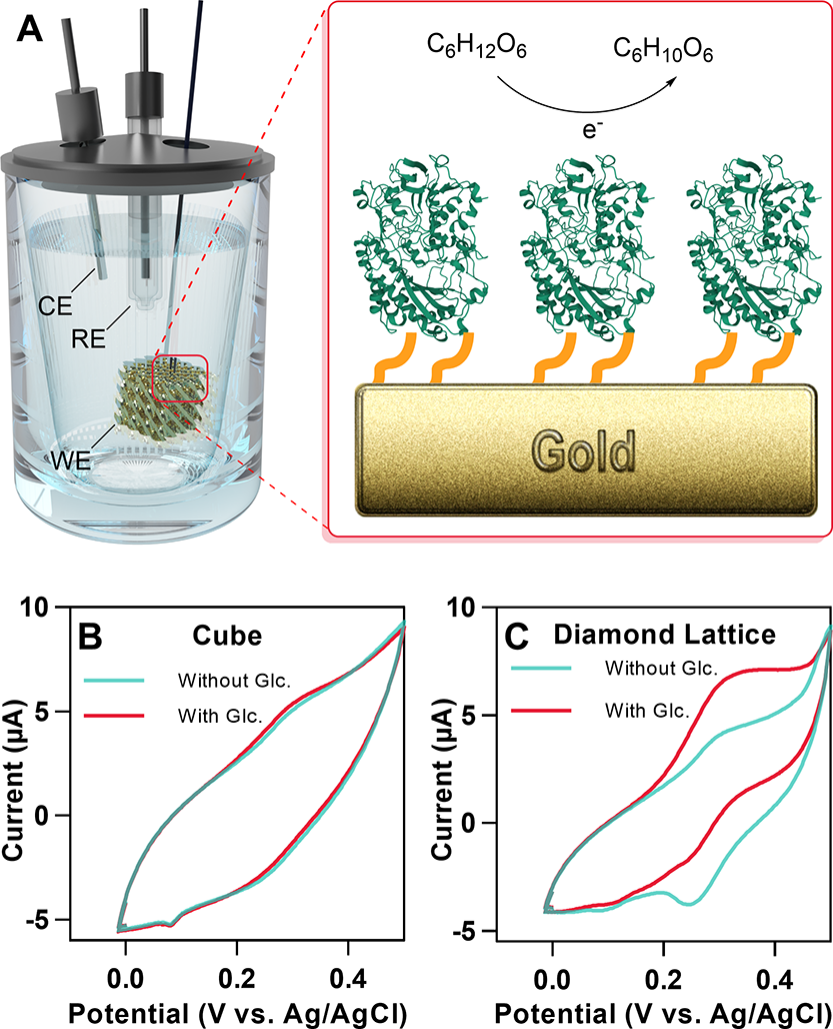
Application of 3D printed microelectrodes as an enzymatic anode: (A) schematic representation of the enzymatic glucose oxidation process inside an electrochemical cell, including the counter electrode (CE), reference electrode (RE), and 3D printed gold working electrode (WE). Cyclic voltammetry curves of glucose oxidation with microelectrodes of (B) cube and (C) diamond lattice form.

As a proof-of-concept, we examined glucose oxidase (GOx)-immobilized 3D gold microelectrode as a potential bioanode for a single enzyme biofuel cell (Figure 6A). The enzyme immobilization protocol consists of surface functionalization of a 3D microstructured gold electrode with a self-assembled monolayer (SAM) of cysteamine, incubation for 1 h with GOx solution, washing with PBS buffer, and then glutaraldehyde cross-linking treatment for absorbed GOx immobilization and stabilization on the electrode. The activity of GOx was determined by recording the increase in absorbance at 414 nm produced by the oxidation of the ABTS (ε = 36,000 M^–1^ cm^–1^ at 414 nm) using a spectrophotometer thermoregulated at 25 °C with magnetic stirring. Figure S12 shows that 80% of the initial enzyme activity remained in the solution. This means that 10 μg of the enzyme, corresponding to 20% of the initial activity in the solution, was immobilized on the gold lattice electrode, confirming the good stability of the immobilized enzyme.

Cyclic voltammetry (CV) was used to study the electrocatalytic activity of GOx in the presence of glucose across the electrode surface of the 3D printed bioanodes. Among three TPMS lattice geometries (diamond, gyroid, and primitive with surface areas of 64.4, 54.2, and 42.2 mm^2^, respectively),^42^ we measured the electrochemical activities of a 3D diamond lattice with the highest surface area and a simple cube-shaped electrode with the smallest surface area (15 mm^2^, no internal pores) to investigate the effect of the increased catalytic surface area on the electrocatalytic activity. This analysis was carried out in PBS (100 mM, pH 7) solution saturated with nitrogen gas at a concentration of 50 mM glucose solution and 0.5 mM ferrocenemethanol as a redox mediator. The mediator was used to facilitate the electron transfer between the enzyme and electrode by accepting the electrons from the oxidation of glucose to glucolactone catalyzed by GOx and subsequently becoming oxidized at the electrode (Supporting Information, Scheme S1).^47^ N_2_ bubbling was used to minimize a possible O_2_ interference as electron acceptor in the enzymatic electrocatalytic reaction, which would diminish the electrochemical signal. The 3D printed cube bioanode exhibited very weak electroenzymatic activity in the presence of 50 mM glucose, as shown in Figure 6B. The 3D printed diamond lattice bioanode, on the other hand, demonstrated a clear mediated electrocatalytic glucose oxidation current observed by the increase of the ferrocenemethanol oxidation wave, while its reduction wave disappeared (Figure 6C). The catalytic current of 2.5 μA at 0.35 V was measured per 2 mm^3^ of the diamond lattice microelectrode, which is almost 10 times higher than 0.27 μA of the cube microelectrode with the same dimension. This demonstrates that the higher catalytic surface area of the 3D diamond lattice electrode leads to the generation of higher current output compared to the cube microelectrode with a small surface area. The current responses observed in the cyclic voltammogram without glucose in Figure 6C correspond to the oxidation and reduction of the redox mediator (ferrocenemethanol) in the solution. The peak current is higher in the diamond lattice microelectrode compared to the cubic electrode (Figure 6B,C). This suggests that the diamond lattice electrode has a larger electroactive surface area than the cubic electrode. The microstructure of the diamond lattice would favor radial diffusion of the redox mediator to the electrode surface, thus enhancing the faradaic currents (peak currents) compared to the capacitive current (background current). The obtained results demonstrate that the combined advantages of high conductivity and large catalytic surface area of the 3D printed gold microelectrode significantly increased the enzymatic anode efficiency for glucose oxidation and consequently enhanced the current output. In addition, the gold surface of the 3D microstructured bioanode remained the same during the cyclic voltammetry experiment, demonstrating the strong interfacial adhesion of the gold surface during bioelectrocatalysis. Our methodology can be applicable to metallization of porous electrode substrates with micro- and mesopores, particularly porous polymer template assisted nanostructures, to achieve robustness of electrodes and high enzymatic bioelectrocatalysis.^48^

## 3 Conclusions

This work demonstrated a method to fabricate robust 3D conductive microstructures using PμSLA coupled with electroless plating. The poor adhesion between a metal and a polymer, one of the main issues with electroless plating, was solved by introducing a thiol interfacial adhesion layer on the 3D printed polymer surface. The thiol groups on the polymer surface provide strong bonding with gold during electroless plating, confirmed by surface and interfacial analysis by XPS, FIB-SEM, EDX, and ToF-SIMS, leading to uniform gold layer formation and thus high electrical conductivity. An adhesion test conducted on the 3D conductive gold samples demonstrated that nearly all deposited gold remained intact after the adhesion test, and the samples were highly conductive (2.2 × 10^7^ S/m), proving significantly improved interfacial adhesion. This method enables interface engineering between the polymer and the deposited gold layer to achieve good adhesion. An initial assessment was performed to validate the electrochemical performance of the 3D microelectrode as an enzymatic anode for use in an enzymatic biofuel cell. High performance of EFC based on the 3D printed lattice-structured gold microelectrode was demonstrated by the increased catalytic surface area compared to a simple cubic electrode. The work opens up the possibility of employing the proposed method not only for the improvement of the EFCs but also for applications in implantable energy supply devices and self-powered biosensors.

## 4 Experimental Section

### 4.1 Materials

Irgacure819 was obtained from BASF. All other chemicals, including pentaerythritol tetraacrylate, 3-mercaptopropyltrimethoxysilane (95%), hexylamine (99%), ethanol, hydrochloric acid (0.1 M), hydroxylamine hydrochloride curcumin, glucose oxidase from *Aspergillus niger*, cysteamine, glutaraldehyde grade I, 25% in H_2_O solution, d-(+)-glucose, ferrocene methanol, and 2,2′-azino-bis(3-ethylbenzothiazoline-6-sulfonic acid) (abts), and the dielectric polymer paste were purchased from Sigma-Aldrich and Sun Chemical, respectively, and were used without further purification. Horseradish peroxidase (268 U/mg of protein) was acquired from Fisher Scientific Spain (Madrid, Spain). Silver paint (RS PRO Conductive Lacquer) was purchased from RS Components. All aqueous solutions were prepared using deionized (DI) water. At least three replicates of each sample’s preparation and analysis were performed to ensure the protocol’s reproducibility, and error bars were included in the statistical analysis to show the standard deviation.

### 4.2 Synthesis of Multifunctional Acrylate Monomer

The multifunctional acrylate monomer (MP monomer) was prepared by modifying the literature procedure.^26^ Briefly, equal moles of PETA (1.0 equiv, 3 g, 8.51 mmol) and MPTMS (1.0 equiv, 1.5 mL, 8.51 mmol) were stirred vigorously for 5 min. Hexylamine (0.1 equiv, 0.111 mL, 0.85 mmol) was then added slowly, and the mixture was stirred overnight at 40 °C.

^1^H NMR (500 MHz, CDCl_3_) δ 6.45–6.35 (m, 2H), 6.15–6.06 (m, 2H), 5.91–5.82 (m, 2H), 4.31–4.09 (m, 8H), 3.71–3.63 (m, 1H), 3.56 (s, 9H), 3.52–3.42 (m, 1H), 2.80–2.68 (m, 3H), 2.66–2.50 (m, 4H), 1.74–1.65 (m, 2H), 1.63 (s, 1H), 1.26 (d, J = 15.3 Hz, 1H), 0.74 (dd, J = 9.7, 6.8 Hz, 2H). ^13^C NMR (126 MHz, CDCl_3_) δ 131.67, 127.48, 62.30, 50.43, 41.98, 34.50, 26.52, 22.76, 8.42.

### 4.3 Preparation of the Photocurable Resin

The photocurable resin was prepared by adding curcumin of 0.5 wt % as a photoabsorber to the MP monomer. The mixture was stirred at 60 °C overnight. Then, Irgacure 819 of 1 wt % as a photoinitiator was added to the mixture. The reaction container was wrapped with aluminum foil to prevent photoreaction. The resin was stirred at room temperature for 12 h before printing.

### 4.4 Degree of Polymerization

The degree of polymerization (DP) was determined by Fourier transform infrared spectroscopy (FT-IR 4200, Shimadzu Co., Kyoto, Japan). Irgacure 819 was added to the MP monomer at different concentrations (0.5, 0.75, 1.0, 1.25, 1.5, and 1.75 wt %). The prepared photocurable resin was placed in a 40 μL standard aluminum sample pan and then photopolymerized using a UV lamp (396 nm) for 2 s. FTIR spectra of cured polymer and uncured resin were analyzed to calculate DP based on the peak areas of the unsaturated C=C bond at 1650 cm^–1^ and a carbonyl group (C=O) at 1730 cm^–1^ using eq 1.
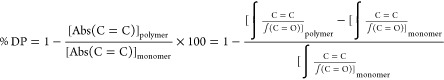
1

### 4.5 3D Printing of Microstructures

A projection micro-stereolithography (PμSLA) system (nanoArch S130 by Boston Microfabrication (BMF) Precision Technology Inc.) was used to manufacture 3D polymeric microstructures. First, 3D lattice models with different surface areas and porosities were generated by the AutoCAD software and digitally sliced into multiple layers with a 10 μm thickness of a single layer using the BMF slicing software. Photopolymerization in the PμSLA system was performed under UV laser exposure with an intensity of 80 mW/cm^2^. The exposure time for the first layer was set at 10 s to achieve good adhesion of a printed sample on the stage. The remaining layers were built with an irradiation time of 2 s and delay time of 5 s for each layer. After printing, the sample was washed with acetone for 30 s to remove the residual resin. The 3D printed sample was then submerged in ethanol overnight to remove unreacted monomers trapped inside the polymer matrix.

### 4.6 Evaluation of the Spatial Resolution of the 3D Printed Structure

The spatial resolution of the 3D printed structure was evaluated by quantifying the overpolymerization (OP) of a bridge-shaped model structure in the *z*-direction using an optical microscope and the ImageJ software. The OP was calculated using eq 2.^34^
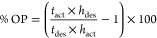
2where *t*_act_ is the thickness of the printed overhang, *t*_des_ is the thickness of the designed overhanging (0.2 mm), *h*_act_ is the height of the printed bridge, and *h*_des_ is the height of the designed bridge (0.84 mm) in Figure S2.

Angular accuracy was determined by comparing the designed component’s angle to the printed part’s angle using the model structure of body-centered cubic (BCC) lattice (Figure S3C,D) with a dimension of 2 mm^3^. The angle of the printed part was measured from the scanning electron microscope (SEM) image using the Autodesk Netfabb 2021 software. The following equation was used to calculate the angular precision:
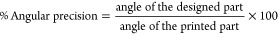
3

### 4.7 Thiol-Functionalization of 3D Polymer Structures

The sol–gel method was utilized to introduce thiol groups on the surface of the 3D printed structure by following the protocol described by Jia et al.^49^ Briefly, the mixture of MPTMS with water at a 1:4 volume ratio, 10% (v/v) of ethanol, and 3.3% (v/v) of 0.1 M hydrochloric acid was stirred at room temperature for 30 min. The resulting mixture was stored under ambient conditions for 2 h. The 3D printed polymer structure was submerged in the prepared MPTMS solution for surface modification. The reaction time from 1 h to 3 days was investigated to find the optimal reaction time for introducing the highest thiol functional groups. The sample was then immersed in ethanol for 12 h to ensure that unreacted MPTMS molecules were removed entirely.

### 4.8 Electroless Gold Plating

The electroless gold plating process began with a seeding step that involved immersing the thiol functionalized polymer (SG-MP) microstructures in the gold nanoparticle solution (5.5 × 10^13^ particles/mL) for 30 min and then sonicating the solution for 1 min in degas mode to remove any trapped bubbles within the 3D printed lattice structure. The sample was thoroughly washed with water three times using a bath sonicator in the delicate mode to remove unattached gold nanoparticles. After the seeding step, the sample was submerged in 0.5 mL HAuCl_4_·3H_2_O solution (5 mg/mL) and sonicated for 10 s in degas mode. The electroless plating process was initiated by adding 0.5 mL of hydroxylamine solution (40 mg/mL) as a reducing agent and sonicating the solution in a delicate mode. The electroless plating reaction was completed within 7 min. The gold-coated microstructure was then washed with deionized water three times.

### 4.9 Enzyme Immobilization

The immobilization protocol used was found to be the best in terms of activity/stability for the immobilization of GOx on glutaraldehyde-amino-agarose.^50^ To carry out enzyme immobilization, the gold surface was first functionalized by immersing it in a 1 M solution of cysteamine overnight while shaking it on the roller. After thorough washing, the electrode was placed in a PBS buffer solution containing 5 μg/mL of the GOx enzyme in 5 mM PBS for an hour. The electrode was then washed to remove unimmobilized enzymes. The electrode was then immersed into 1% (v/v) glutaraldehyde diluted in 50 mM of PBS at pH 7.0, and the mixture was shaken for an hour to modify the amino groups on the enzyme and the support and permit the reaction between these groups located in the enzyme and the gold. The immobilized enzyme was then washed and resuspended overnight in PBS pH 8 (100 mM) to maximize the enzyme–support reaction. The amount of immobilized enzyme was estimated by comparing the activity of the enzyme solution before and after the enzyme immobilization process according to Betancor et al.^50^

### 4.10 X-ray Photoelectron Spectroscopy (XPS)

The samples were analyzed using the Kratos AXIS ULTRA with a monochromatic Al Kα X-ray source (1486.6 eV) operated at 10 mA emission current and 12 kV anode potential (120 W). The spectra were acquired with the Kratos VISION II software. A charge neutralizer filament was used to prevent surface charging. The survey spectra (binding energy range from 1400 eV to −5 eV) were acquired at a pass energy of 80 eV, step of 0.5 eV, and sweep time of 20 min and used to estimate the total atomic % of the detected elements. The high-resolution spectra at a pass energy of 20 eV, step of 0.1 eV, and sweep time of 10 min were also acquired for photoelectron peaks from the detected elements. The spectra were charge corrected to the C 1s peak (adventitious carbon) set to 284.8 eV. The Casa XPS software (version 2.3.19 PR1.0) was used for peak fitting and quantification.

### 4.11 Time-of-Flight Secondary Ion Mass Spectrometry (ToF-SIMS)

ToF-SIMS was carried out using a ToF-SIMS IV instrument (IONTOF GmbH). Secondary ion mass spectra were acquired in negative ion polarity mode using a 25 keV Bi_3_^+^ primary ion beam delivering 0.3 pA. The primary ion beam was raster scanned over different areas with the total ion dose kept under the static limit of 10^13^ ions/cm^2^ for surface analysis The ToF analyzer was set with a 200 μs cycle time, resulting in a mass range between 0 and 3490 mass units, and a low-energy (20 eV) electron flood gun was employed to neutralize charge build-up. ToF-SIMS depth profiling was done in dual-beam mode by raster scanning the 25 keV Bi_3_^+^ primary ion beam over a 100 × 100 μm^2^ region at the center of 300 × 300 μm^2^ sputter craters formed using a 5 keV Ar_1400_ gas cluster ion beam (GCIB) delivering 1.5 nA (higher depth resolution for near surface analysis) and 5 keV Ar_1900_ GCIB delivering 12 nA (lower depth resolution for reaching buried interface). The measurement was performed in the “non-interlaced’ mode with a low-energy (20 eV) electron flood gun employed to neutralize charge build-up. Data analysis was done using SurfaceLab 7.1. All ToF-SIMS intensity maps were normalized by total ion counts to correct for topographic features and subsequently normalized by the maximum profile intensity of each secondary ion. Optical profilometry was used to determine crater depths after ToF-SIMS depth profiling experiments and calibrate the depth scale in combination with information obtained by FIB-SEM. Scans were obtained using a Zeta-20 optical microscope (Zeta Instruments) in a *Z* range of 4.6 μm. The number of steps was set to 328, allowing for a *z* step size of 14 nm.

### 4.12 Scanning Electron Microscope (SEM)

SEM (Hitachi TM 3030) coupled with an energy-dispersive X-ray (EDX) analyzer was used to examine the printing resolution, uniformity of gold layer, and chemical composition of 3D gold-deposited polymer microstructures.

### 4.13 Focused Ion Beam Scanning Electron Microscopy (FIB-SEM)

FIB-SEM (Zeiss Crossbeam 550, Carl Zeiss, Germany) was used to create and examine the cross sections of the conductive 3D gold microstructures and measure the thickness of the coated gold layer. In the microscope, the sample was tilted at an angle of 54°. The Ga^+^ beam was used to dig a rectangle (20 μm × 5 μm) at 30 nA, and this was improved by lower current milling at 1.5 nA to give a smoother finish at the cut face. During SEM imaging, the in-lens, SESI, and backscattered detectors were operated at 2 kV to acquire images of cross sections.

### 4.14 Mechanical Adhesion Test

The mechanical adhesion between the deposited gold and the polymer substrate was measured by sticking a high-performance clear 3M scotch tape to a 3D printed metallized polymer and then sharply peeling it off at an angle of 90°. The sample was analyzed with EDX mapping before and after the tape test to measure the adhesion of the deposited gold (Figure 4B). From EDX mapping images, the area of gold was calculated using the ImageJ software. The adhesion of the deposited gold was determined using the following equation:
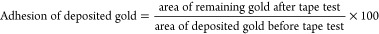
4

### 4.15 Electrical Conductivity Measurement

The conductive 3D gold electrode with a cube structure (2 × 2 × 0.5 mm) was manufactured, and its sheet resistance (*R*_s_) was measured using the Van der Pauw technique.^51^ Horizontal resistance (*R*_H_) and vertical resistance (*R*_V_) were measured using a four-probe micromanipulator system (Micromanipulator, model MM 450 PM) and a sourcemeter (Keithley 2400, Tektronix Inc., Shanghai, China), and *R*_s_ was calculated using eq 5. The average *R*_s_ was taken from three independent measurements.

The conductivity (σ) of the deposited gold was calculated using eq 6, where *t* is the thickness of the gold film acquired from FIB-SEM analysis.
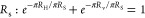
5
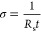
6

### 4.16 Enzyme Activity Determination

The activity of GOx was determined by recording the increase in absorbance at 414 nm produced by the oxidation of the ABTS (ε 414 = 36,000 M^–1^ cm^–1^ under these conditions) using a V-730 Jasco (Madrid, Spain) spectrophotometer thermoregulated at 25 °C with magnetic stirring. The ABTS assay was performed using 1.8 mL of 100 mM of PBS at pH 7.0 containing 0.5 mL of d-glucose at 1 M, 100 μL of ABTS at 10 mg/mL prepared in 100 mM of PBS at pH 7.0, and 50 μL of horseradish peroxidase at 0.1 mg/mL prepared in 100 mM of PBS at pH 6.0. It was checked that the activity values were maintained if using half or double of peroxidase, confirming that the activity depends only on the amount of glucose oxidase. The reaction starts when 50 μL of the solution was added. One unit (U) of activity was defined as the amount of enzyme that oxidizes 1 μmol of substrate per minute under the specified conditions.^50^

### 4.17 Electrochemical Characterization

All electrochemical characterizations were performed using an Autolab PGSTAT30 potentiostat/galvanostat from Metrohm Autolab (Utrecht, The Netherlands) employing a three-electrode setup with a bioanode electrode as a working electrode, a platinum mesh as a counter electrode, and the Ag/AgCl (3 M KCl) as a reference electrode. To prepare the working electrode, an insulated copper wire was connected to the gold-coated electrode using a silver paint and insulated with a dielectric polymer before the enzyme immobilization process. The electrodes were immersed in phosphate buffer saline (PBS) (100 mM) at pH 7.0. Prior to the characterization of the anode, N_2_ was bubbled into the electrochemical cell for 10 min. Ferrocene methanol (0.5 mM) and glucose (50 mM) were then added to the buffer as a mediator and a substrate, respectively. Cyclic voltammetry (CV) was used for electrochemical analysis at a scan rate of 10 mV/s. All potentials are presented in relation to the standard hydrogen electrode (SHE).

## Supporting Information Available

Thiol–Michael reaction mechanism of the MP monomer, degree of photopolymerization, 3D design and printed bridge for overpolymerization analysis, XPS results of MP, ToF-SIMS mapping, EDX of MP cubes for adhesion test, EDX of the lattice cross section, FIB-SEM comparison of gold-coated samples, XPS results of SG-MP before and after electroless plating, and UV–vis spectroscopy results of enzyme immobilization on the microelectrode (PDF)
