# Supplementary material for: Additively Manufactured 3D Micro-bioelectrodes for Enhanced Bioelectrocatalytic Operation
Source: ACS Appl Mater Interfaces. 2023 Mar 10;15(11):14914–24. doi: 10.1021/acsami.2c20262 (PMC10037242; doi:10.1021/acsami.2c20262)
Supplement: Supplementary file 1 — am2c20262_si_001.pdf [file am2c20262_si_001.pdf]

# Supporting Information
for

# Additively Manufactured 3D Micro-Bioelectrodes for Enhanced Bioelectrocatalytic Operation

*Keyvan Jodeiri,[a] Aleksandra Foerster,[a] Gustavo F. Trindade,[a,b] Jisun Im,[a] Diego Carballares,[c] Roberto Fernández-Lafuente,[c,d] Marcos Pita,[c] Antonio L. De Lacey,[c] , Christopher D Parmenter,[e] and Christopher Tuck, [a]**

*[a]Centre for Additive Manufacturing, Faculty of Engineering, University of Nottingham, University Park, Nottingham, NG7 2RD, United Kingdom*

*[b]National Physical Laboratory, Hampton Road, Teddington, TW11 0LW, United Kingdom*

*[c]Instituto de Catálisis y Petroleoquímica, CSIC, C/Marie Curie 2, 28049 Cantoblanco, Madrid, Spain*

[d]*Center of Excellence in Bionanoscience Research, Member of the external scientific advisory board, King Abdulaziz University, 21589 Jeddah, Saudi Arabia*

[e]*Nanoscale and Microscale Research Centre, University of Nottingham, University Park, Nottingham, NG7 2RD, United Kingdom*

*Email: Christopher.Tuck@nottingham.ac.uk

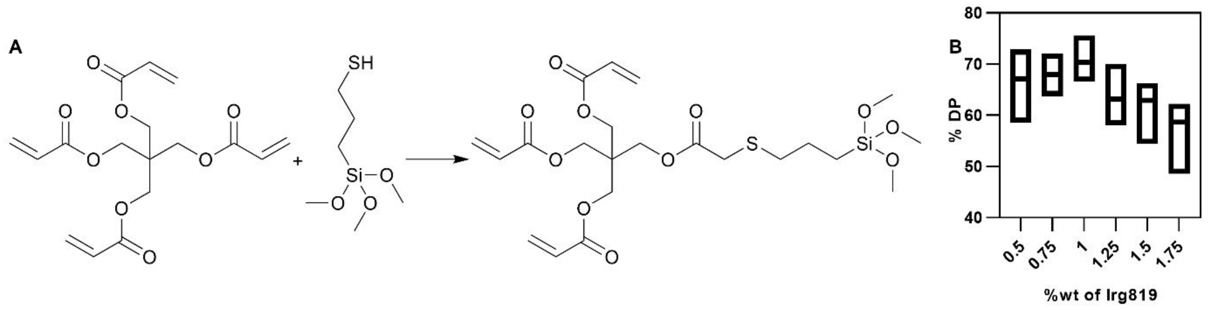


Figure S1. Preparation of photocurable resin. (A) Thiol-Michael addition reaction between PETA and MPTMS. (B) Degree of polymerization (DP) measured at different photoinitiator concentrations, Irgacure 819.

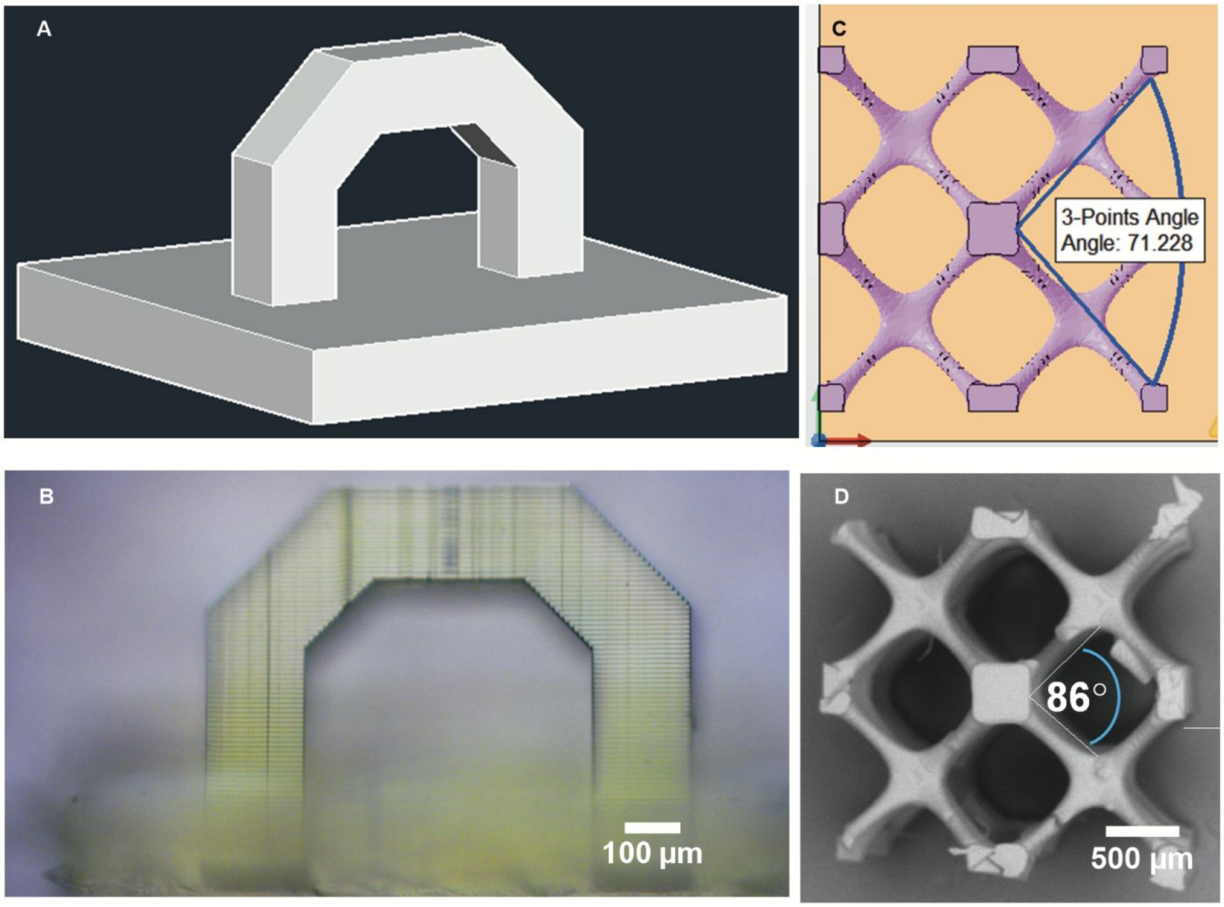


Figure S2. Bridge shaped 3D model structure used to calculate over-polymerization. (A) the designed 3D model. (B) 3D printed bridge microstructure using PμSLA, (C) Designed angle of the BCC model, and (D) Measured angle of the printed structure.

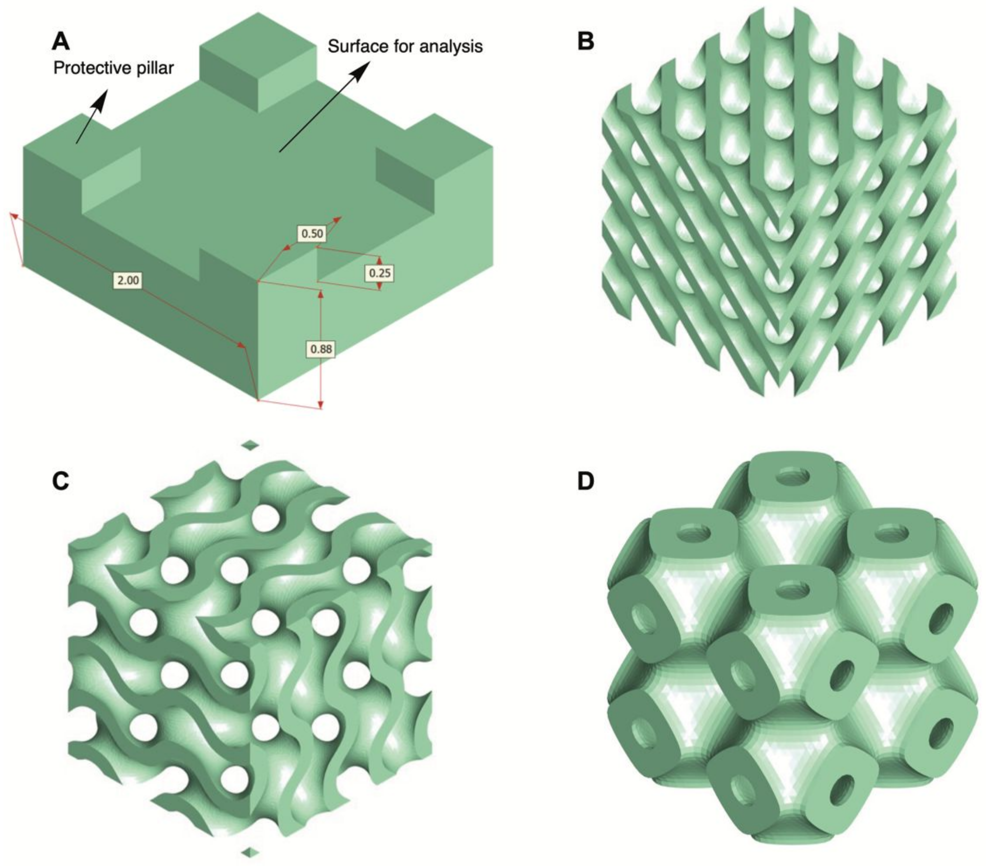


Figure S3. 3D model structures used for the fabrication of 3D microelectrodes: (A) Cube structure (2mm × 2mm × 0.5mm) Four pillars at the corners of the cube structure were designed to prevent surface damage that might be induced during sample handling and all surface characterization was performed on the center surface of the sample with pillars. (B) Diamond, (C) Gyroid, and (D) Primitive lattice structures with the dimension of $2mm^3$.

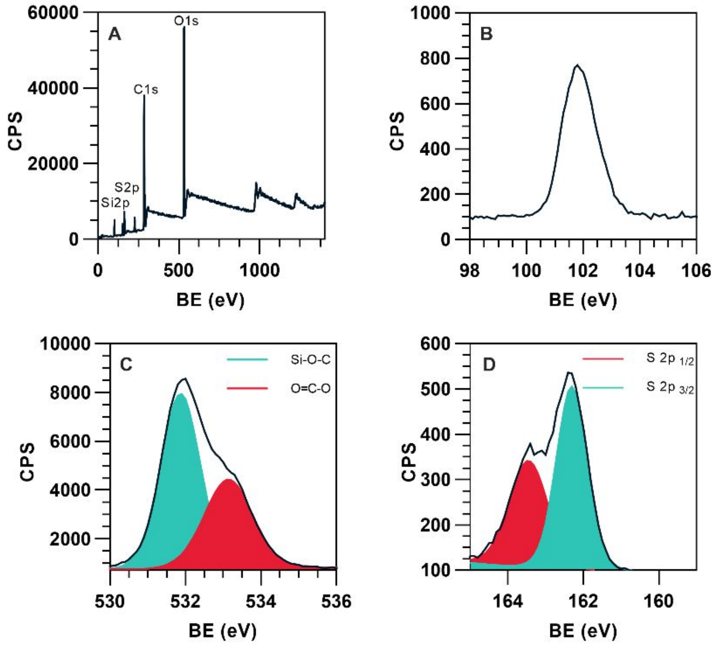


Figure S4. XPS analysis on 3D printed MP polymer. (A) Survey scan spectrum. High-resolution spectra of (B) Si2p, (C) O1s, and (D) S2p.

Table S1. Atomic percentages of the elements (at%) obtained from the XPS spectra of 3D printed polymer structures before (MP polymer) and after functionalisation (SG-MP polymer prepared for 3 days reaction).

| Sample | Element (at%) | | | |
|---|---|---|---|---|
| | C 1s | O 1s | S 2p | Si 2p |
| MP | 68.30 | 25.95 | 2.45 | 3.30 |
| SG-MP | 60.24 | 24.73 | 6.64 | 8.39 |

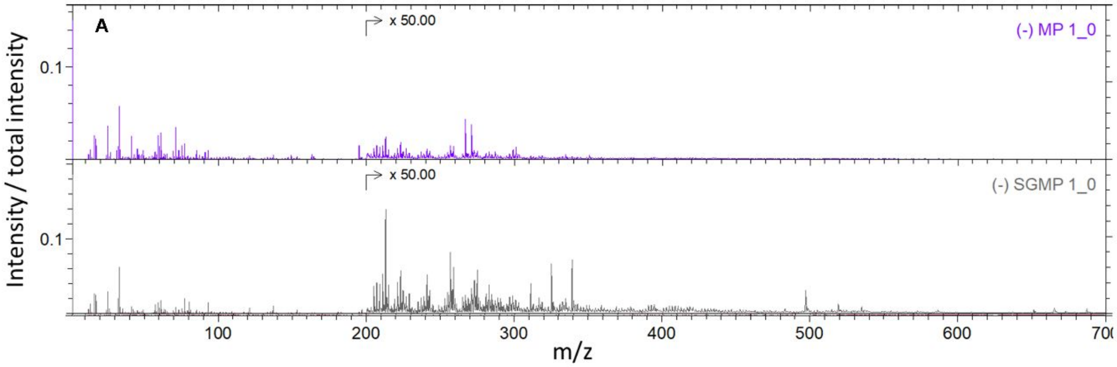


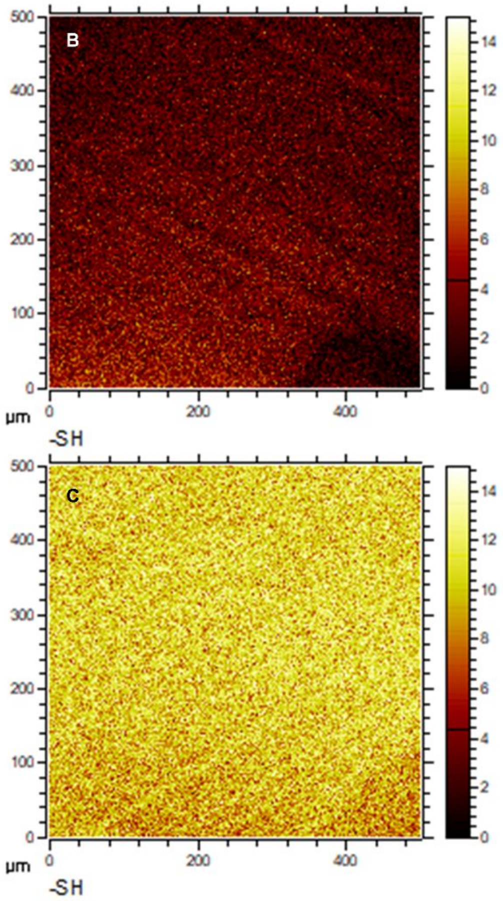


| Centre Mass (u) | Assignment |
|---|---|
| 24.00 | C2- |
| 32.98 | HS- |
| 55.97 | C_2S- |
| 71.02 | C_3H_3O_2- |
| 59.97 | SiO_2- |
| 25.00 | C_2H- |
| 15.99 | O- |
| 31.97 | S- |
| 43.97 | CS- |
| 55.02 | C_3H_3O |

Figure S5. Surface chemical composition analysis of 3D printed polymers before (MP polymer) and after surface functionalisation with MPTMS (SG-MP polymer). (A) ToF-SIMS spectra of MP polymer and SG-MP polymer. ToF-SIMS mapping data of (B) MP polymer and (C) SG-MP polymer. (D) Peak assignment in ToF-SIMS spectra.

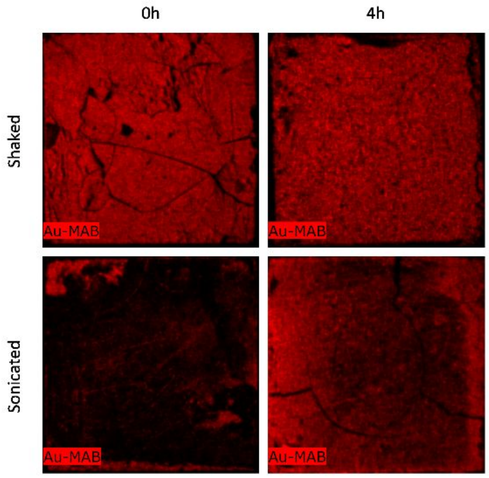


Figure S6. Comparison of gold coated sample during the shaking and sonication.

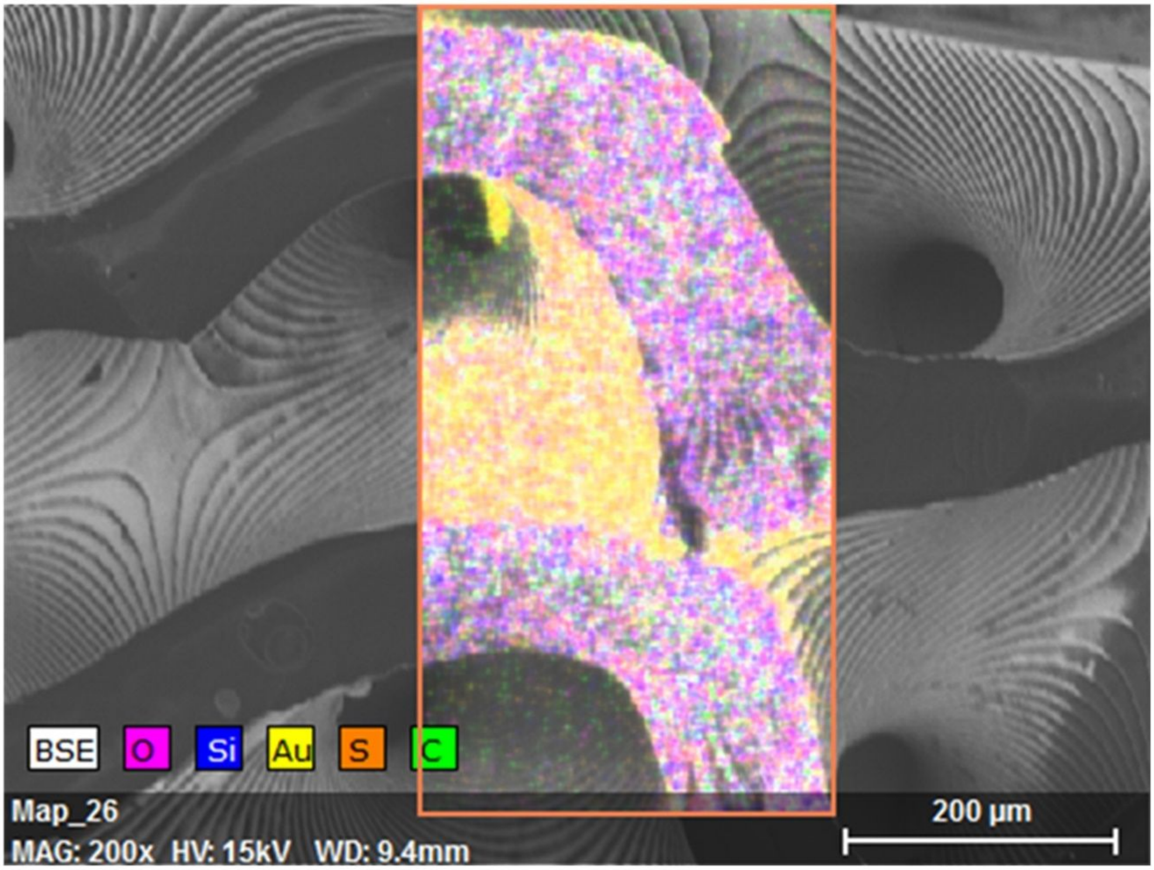


Figure S7. EDX mapping data of the cross-section of gold-coated diamond lattice structure indicating the unform gold deposition inside the lattice structure.

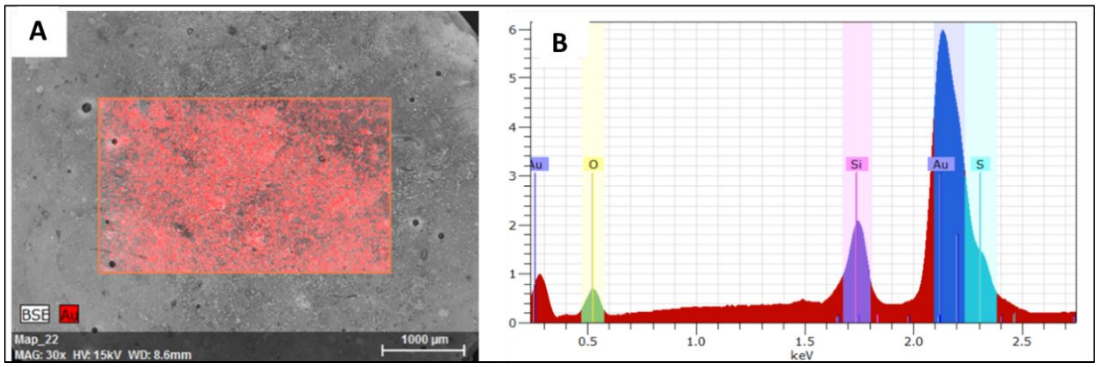


Figure S8. Surface chemical composition of gold-coated SG-MP (*$t_{func}$=3h)* polymer using EDX. (A) SEM image showing EDX mapping signal of Au shown in red. (B) EDX spectrum proving the existence of Au, O, Si, and S.

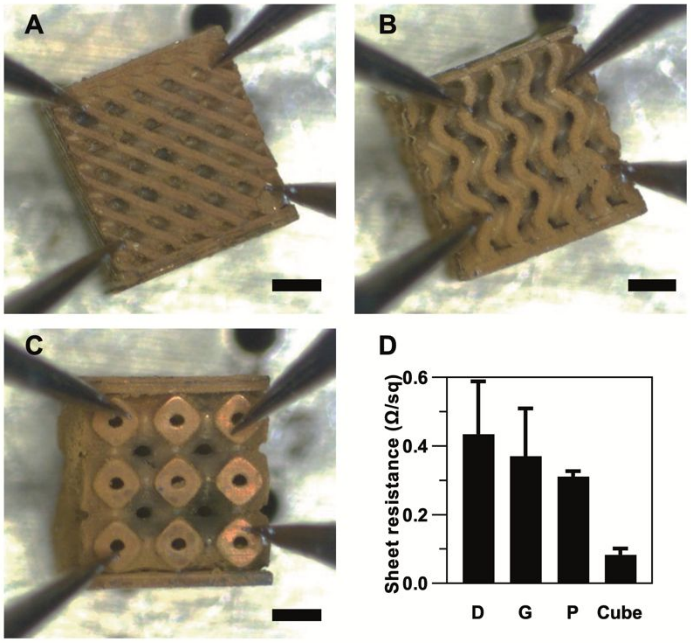


Figure S9. Sheet resistance measurement of 3D gold microelectrodes using van der Pauw method: optical microscope images of gold-coated (A) diamond, (B) gyroid, and (C) primitive lattice

electrodes. (D) Sheet resistance of four 3D gold microelectrode structures. D, G, and P stand for diamond, gyroid, and primitive lattices, respectively. The scale bar is 500 μm.

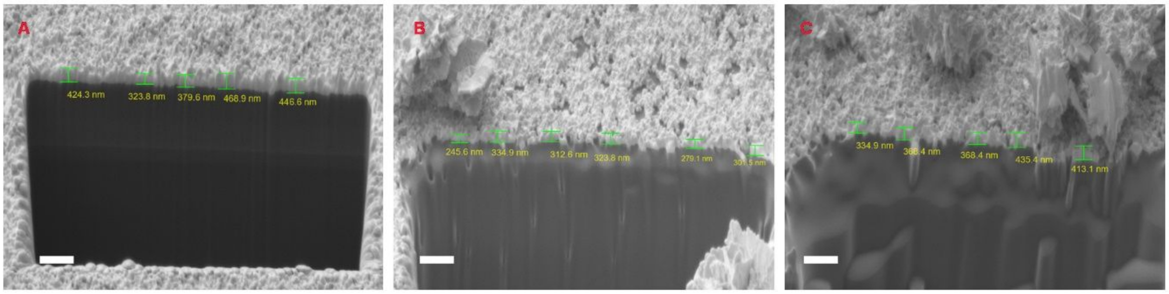


Figure S10. Thickness measurement of the deposited gold on 3D printed microstructures using FIB-SEM. Cross-section images of the electroless gold plated samples prepared from the SG-MP polymers with (A) 4 hours, (B) 1 day and (C) 2 days functionalisation time. The scale bars are 1 μm. Cross-section measurements were made in a minimum of 5 places (as shown) following FIB-milling. The automated tilt-correction algorithm was applied in the Zeiss SmartSEM software (v6.07) at an angle of 54°.

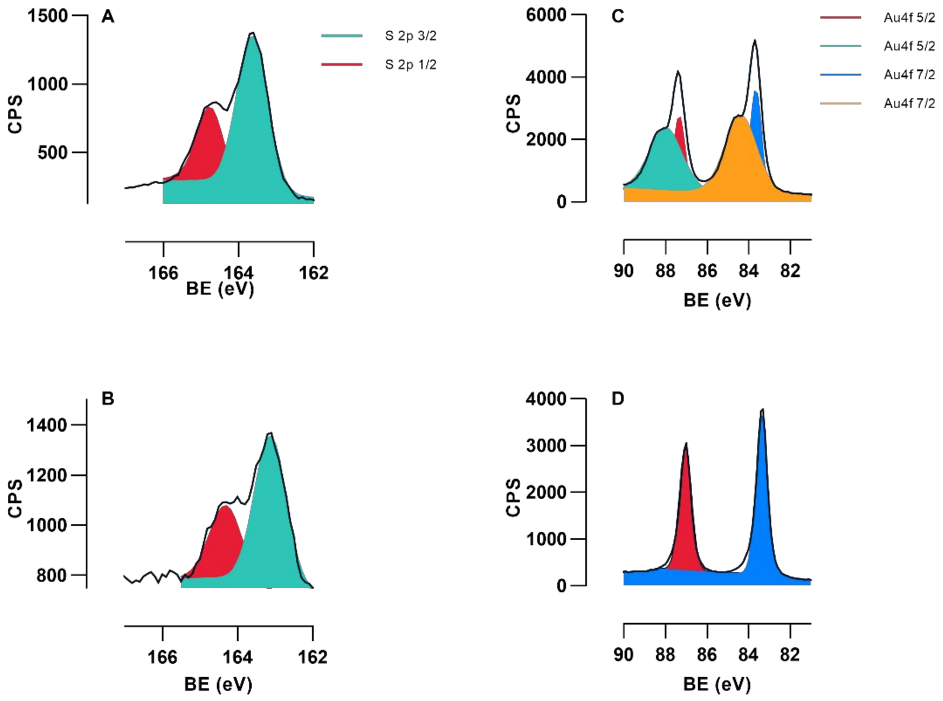


Figure S11. XPS analysis on electroless gold plated polymers with different gold layer thicknesses. High-resolution spectra of S2p core level of (A) the SG-MP polymer at $t_{func}$ = 3h and (B) the SG-MP polymer coated with a thin gold layer. High-resolution spectra of Au4f core level of (C) the SG-MP polymer covered with a thin gold layer and (D) the SG-MP polymer coated with a thick gold layer.

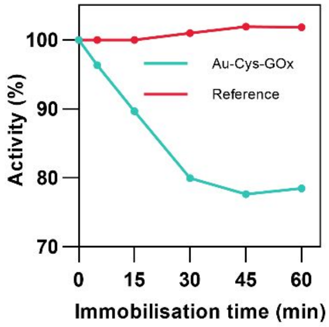


Figure S12. GOx immobilization course on the cysteamine-modified gold electrode that has been functionalized with cysteamine. The data in cyan corresponds to the decrease of the catalytic activity of a 5 μg/mL solution of GOx enzyme in 5 mM PBS solution upon incubation of the electrode (solution aliquots were taken at different times to measure the glucose oxidation activity using ABTS as electron acceptor). The data in red correspond to the equivalent experiment in absence of the modified gold electrode.

***GOx + glucose → GOxH$_2$ + gluconolactone***
***2Fc$^+$ + GOxH2 → 2Fc + GOx + 2H$^+$***
***Fc → Fc$^+$ + e$^-$ (reaction of ferrocenemethanol at the electrode)***

Scheme S1. Electrocatalytic reaction of glucose oxidase in bioanode.
